# Memory reports are biased by all relevant contents of working memory

**DOI:** 10.1038/s41598-024-51595-6

**Published:** 2024-01-30

**Authors:** Paul Zerr, Surya Gayet, Stefan Van der Stigchel

**Affiliations:** 1 Experimental Psychology, Helmholtz Institute, Utrecht University, Utrecht, The Netherlands; 2 Radboud University Medical Center, Donders Institute for Brain, Cognition and Behavior, Nijmegen, The Netherlands

**Keywords:** Computational neuroscience, Visual system, Human behaviour

## Abstract

Sensory input is inherently noisy while the world is inherently predictable. When multiple observations of the same object are available, integration of the available information necessarily increases the reliability of a world estimate. Optimal integration of multiple instances of sensory evidence has already been demonstrated during multisensory perception but could benefit unimodal perception as well. In the present study 330 participants observed a sequence of four orientations and were cued to report one of them. Reports were biased by all simultaneously memorized items that were similar and relevant to the target item, weighted by their reliability (signal-to-noise ratio). Orientations presented before and presented after the target biased report, demonstrating that the bias emerges in memory and not (exclusively) during perception or encoding. Only attended, task-relevant items biased report. We suggest that these results reflect how the visual system integrates information that is sampled from the same object at consecutive timepoints to promote perceptual stability and behavioural effectiveness in a dynamic world. We suggest that similar response biases, such as serial dependence, might be instances of a more general mechanism of working memory averaging. Data is available at https://osf.io/embcf/.

## Introduction

The macroscopic world is highly continuous. The features of our surroundings remain relatively stable within short time frames^1^, and objects tend to move in a predictable way. At the same time, sensory systems do not acquire perfect observations of the world in each instant. Instead, we typically rely on incomplete information from series of short gaze fixations and low-resolution peripheral vision, to form an actionable image of the world. While driving a car, for example, we often estimate the speed and position of other cars from brief glances as we cannot afford to look in the mirror for long periods of time. Even without temporal constraints, perception is characterized by repeated, brief sampling behaviour, as for example indicated by the average human fixation duration of only a few hundred milliseconds between saccades^2^. Yet, we are remarkably effective in our interaction with the world, suggesting the presence of adaptive mechanisms that account for noisy sensory input so that precise behaviour can be produced. There is evidence that individual instances of perception of the same object are automatically stored in working memory^3^. If such memory traces are integrated to serve stable perception and effective behaviour, we would expect that similar items in memory are combined under the (implicit) assumption of being sourced from the same object, while dissimilar items in memory would be repulsed from one another to separate information coming from different objects^4,5^. Together, these observations lead to the prediction that the report of a single memory representation is biased by other concurrently held memoranda. The strength of this bias should then be proportional to the similarity, task-relevance and reliability of the respective memory item.

In multisensory perception research, it is well established that information from different senses is combined in a statistically optimal way to form a more reliable estimate of a world state^6,7^. Averaging over multiple observations of the same object necessarily reduces noise and leads to a more accurate estimate than any single instance of sensory input. The same principle could account for noisy information sampling within a single sensory modality by combining multiple consecutive episodes of sensory input in a principled way to generate an increasingly reliable object representation. Several studies have shown that concurrently memorized visual items bias one another^4,5,8–13^. It remains elusive how, and under what circumstances, memory contents are combined, with some studies observing repulsive biases and other studies observing attractive biases, or both (i.e., biases away from, or toward the other memory item). The present study sets out to test whether concurrently held memory representations are integrated for goal-directed use of the memory content.

One phenomenon that has recently received much attention is serial dependence^14–17^, which could be another example of unimodal information integration within short-term memory. Serial dependence refers to a systematic bias, in which behavioural responses to a stimulus are influenced by previously observed stimuli. For example, the reported orientation of a line on a given trial may be consistently pulled towards the orientation reported on the previous trial. Although the experimental paradigm used to measure serial dependence does not explicitly require observers to keep stimuli from preceding trials in memory, these preceding stimuli necessarily need to be somehow stored in memory in order to affect the current trial. One possibility is therefore that serial dependence, as well the bias in concurrently memorized items reviewed above, reflects a process of information integration in memory. This leads to the prediction that, if multiple consecutive items are memorized, target reports are not only biased by preceding stimuli, but by all memorized stimuli, including those that succeed the target. In this study, we will put this prediction to the test by requiring participants to memorize four consecutive stimuli and measuring how memory reports of any single stimulus are influenced by all other (preceding and succeeding) stimuli.

The view that memory traces are integrated to serve stable perception and effective behaviour leads to specific predictions as to the circumstances under which memory reports should be particularly influenced by concurrently memorized stimuli. We would expect that similar items in memory are integrated (as they are likely to originate from the same object), while increasingly dissimilar items are not integrated (as they are more likely to originate from different objects). We predict that attractive biases in memory reports should be particularly prominent when (1) concurrent memory items are task-relevant, and when (2) concurrent memory items are more similar to the reported item. Task-irrelevant and very dissimilar memory items might even produce repulsive biases. This pattern of results has been observed in a number of studies investigating serial dependence^14–24^.

We made the case that averaging over multiple consecutive instances of perception necessarily leads to a more accurate estimate than any single instance of sensory input. Similarly, the process underlying serial dependence has been described as a predictive mechanism, in which previously encountered information aids current estimates^15,25–27^. In other words, serial dependence may arise from an integration of previously and currently observed information in order to capitalize on the learned stability of the world and ameliorate the unreliability of individual perceptual events^15,19,28–30^. There is indeed evidence that human observers apply knowledge of the statistics of the world in longer timeframes, spanning years, as well as just a few seconds^31,32^. Some researchers have proposed a Bayesian efficient coding model, in which a previous observation provides a prior, which is optimally combined with new incoming sensory information^25–27^. In line with this view, it has been shown using generative modelling of fMRI data, that the influence of a previous stimulus on current stimulus report is strongest when the previous stimulus is represented with higher reliability than the current stimulus^1^. Similarly, when multiple items coexist in short-term memory, a stronger (attractive) bias can be expected when target stimuli are more noisy and when distractor stimuli are less noisy. In other words, we expected that the strength of the bias is proportional to the uncertainty of the reported item.

**Figure 1:**
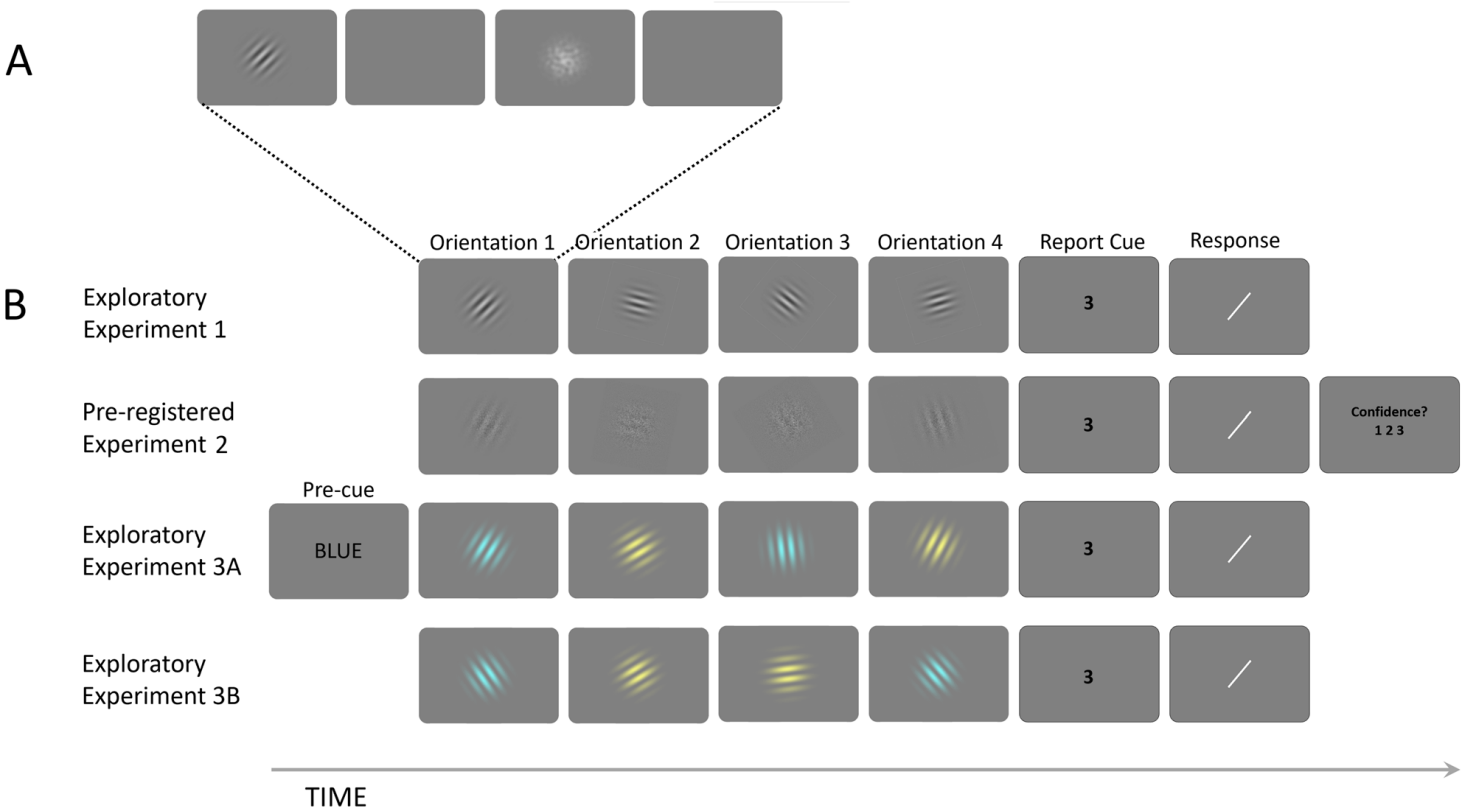
Schematic visualizations of experimental procedures. Sizes and colours are not to scale. (**A**) The sequence of one orientation presentation, consisting of an orientation, a blank, a noise mask and another blank. (**B**) Example trial sequences (excluding blanks and noise masks). In all experiments, observers were sequentially presented with four orientations and were cued to reproduce one of them, by means of an Arabic numeral. The reproduction of the cued orientation was enabled via a rotating white line. See “Methods” for a detailed description. In Experiment 1, standard orientation patches were used to present orientations. In Experiment 2, orientations could be presented as high-noise or low-noise stimuli and observers were additionally asked to rate their confidence in the response via a button press. In Experiment 3A and B, orientations could be presented in one of two colours. In Experiment 3A, the colour of the upcoming target was pre-cued by the word blue or yellow, while in Experiment 3B no pre-cue was presented.

Interestingly, in most studies with concurrently memorized items a repulsive bias is observed^4,5,8–13,33^, whereas sequentially presented stimuli without explicit memory instructions (i.e., serial dependence) tend to elicit an attractive bias, especially for highly similar items^14,15,17–24,34^. These findings seem at odds with our assumption that the biases observed in serial dependence and in concurrent memorization tasks are two expressions of an overarching principle of functionally weighted integration of memory content. One factor that could promote repulsive biases is that memorization of two stimuli for subsequent recognition incites participants to maximally differentiate between these stimuli, whereas memorization of one (or many) stimuli does not^35^. Indeed, nearly all studies to date investigating biases between explicitly memorized items required participants to memorize only two items, perhaps promoting stimulus segregation rather than integration. To circumvent this issue in the present study we required participants to memorize four consecutive stimuli, thus approaching the typical limits of visual working memory capacity. This paradigm uniquely allows us to investigate to what extent memory reports are (differentially) biased toward series of stimuli preceding and succeeding the reported target stimulus (see also Table 1 and Fig. 1).

**Table 1:** Definition of terms used in this article.

Term	Definition
Orientation	Short for: orientation of an oriented sine grating with a Gaussian envelope, also known as Gabor patch
Target	An orientation that was retro-actively cued for explicit report in a reproduction task
Distractor	An orientation presented in the same trial as the target, which needed to be memorized but was not cued for report
Response bias	A systematic deviation of behavioural responses relative to other observed stimuli. Here used to denote the bias under investigation
Serial dependence	The response bias towards (attractive bias) or away (repulsive bias) from a previously observed stimulus. Also known as sequential response bias or visual history effect
Target-distractor difference; ∆	The orientation of a non-target item relative to the target orientation, with negative values indicating that the distractor was oriented counter clockwise relative to the target, and positive values indicating clockwise relative orientation

Taken together, in this study we aim to investigate whether memory reports of memorized consecutive perceptual snapshots are biased towards one-another, in a manner that serves goal-directed, memory-driven behaviour**.** Specifically, we predict that memory reports should be attracted toward task-relevant, similar, and less noisy stimuli that are concurrently memorized. Moreover, if these biases indeed occur within short-term memory (and not during perception or encoding), target reports should be influenced not only by stimuli presented *before* the target, but also by stimuli that are presented *after* the target has already disappeared (i.e., after encoding was completed).

## Results

If consecutively presented stimuli are integrated within short-term memory (rather than during perception or encoding), this would predict that responses are biased not only by items encoded prior to the memory target, but also by items encoded after offset of the memory target. To address this possibility, we tested whether the reported orientation of a retro-actively cued target was influenced by the preceding or succeeding items in a sequence of four items. Specifically, we measured the extent to which target report was biased towards or away from each of the three non-target items (i.e., distractors) in the sequence.

**Figure 2:**
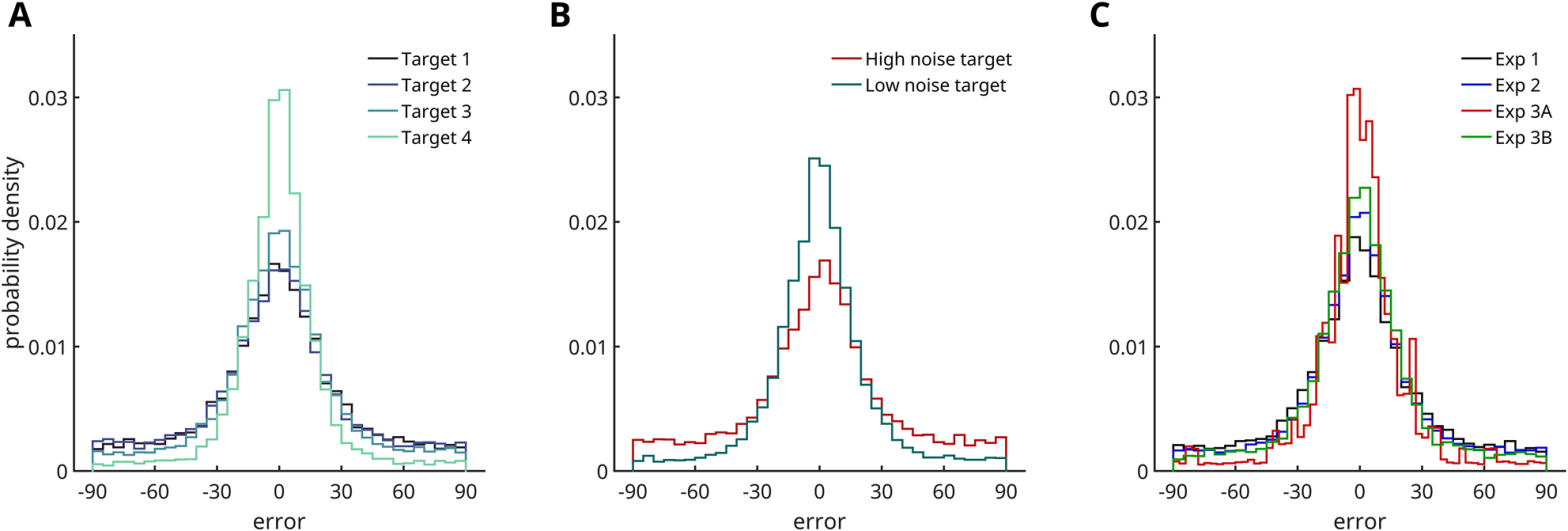
Histograms and density functions of response errors (**A**) per target position (Exp. 1 + 2 + 3B), (**B**) per target noise level (Exp. 2), and (**C**) per experiment. The density of the histograms follow the expected patterns in the data: the most recently shown target (Target 4), low-noise targets and targets in Experiment 3A (smaller memory set size) were remembered with higher precision on average.

**Figure 3:**
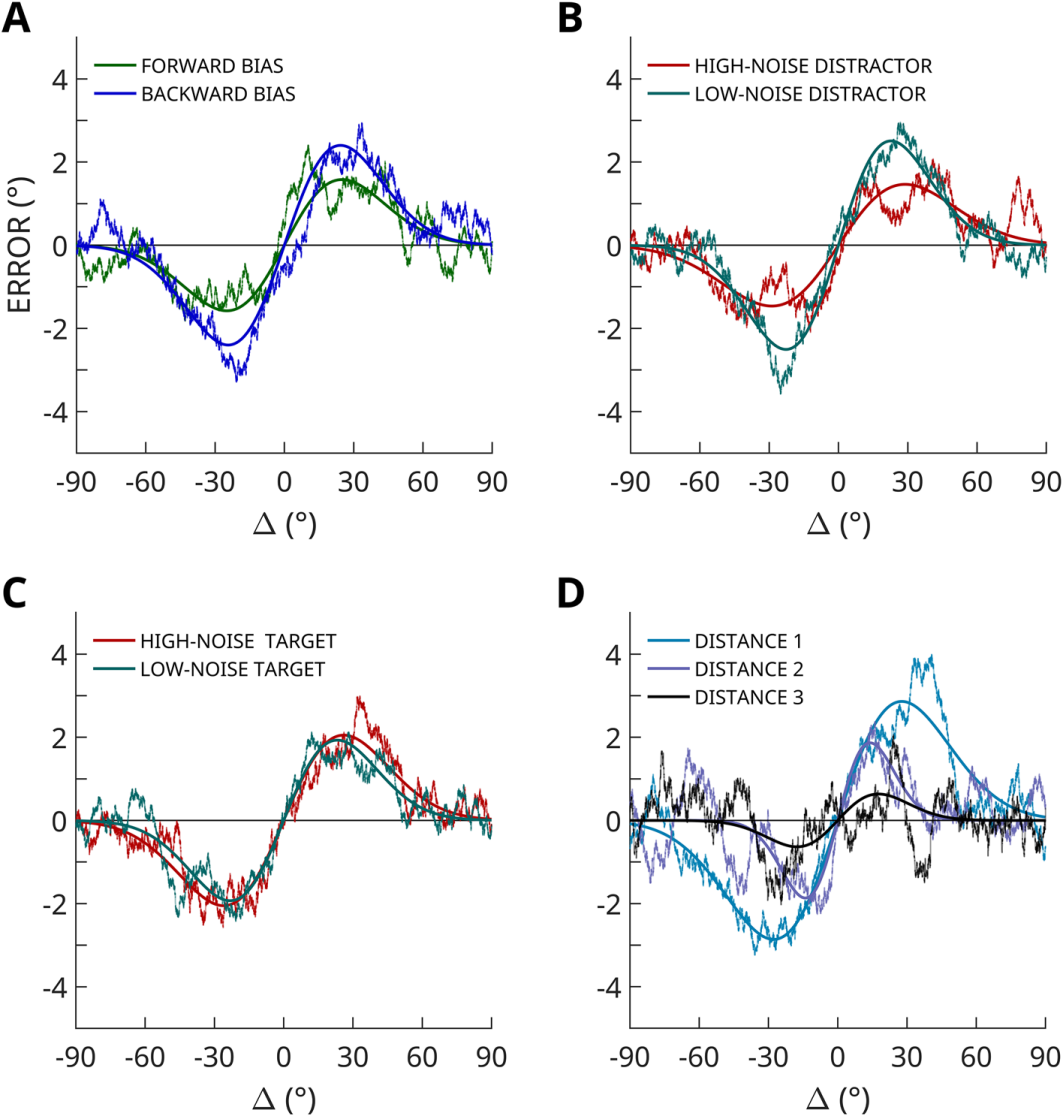
Results of pre-registered Experiment 2. (**A**) Forward (distractor shown before target) and backward (distractor shown after target) response bias obtained from sorting response errors as a function of the orientation difference between the target and a given distractor. Ragged lines represent moving averages over response errors. Smooth lines represent a derivative of Gaussian (DoG) function fit. (**B**) Response bias for high-noise and low-noise distractors. (**C**) Response bias for high-noise and low-noise targets. (**D**) Response bias for distractors one, two or three items removed from the target in the sequence.

**Figure 4:**
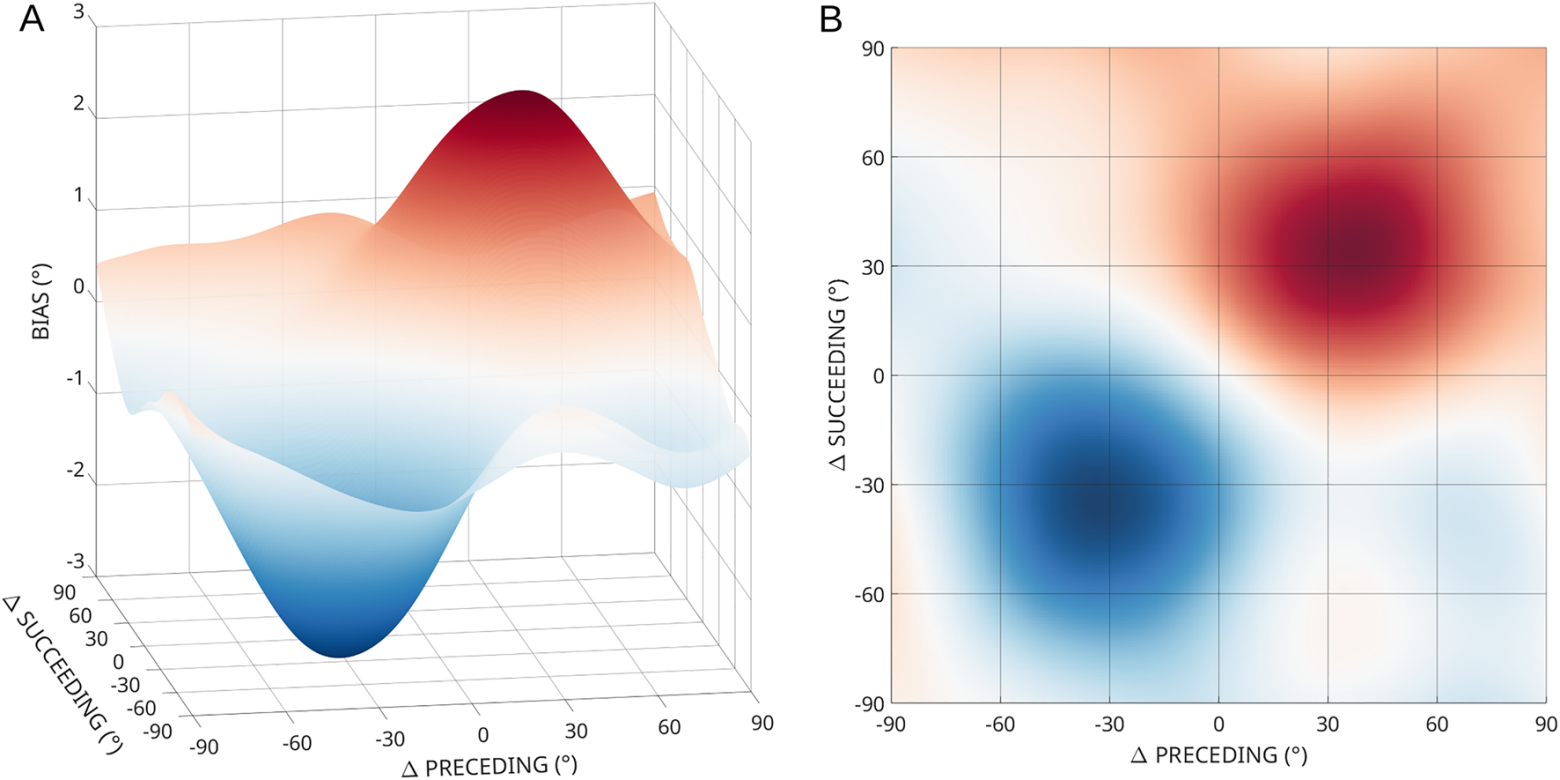
3D surface graph (Lowess fit), illustrating how the effects of preceding and succeeding distractors combine linearly to influence target report (data from Experiments 1, 2 and 3B). (**A**) 3D view. (**B**) Top-down view.

**Figure 5:**
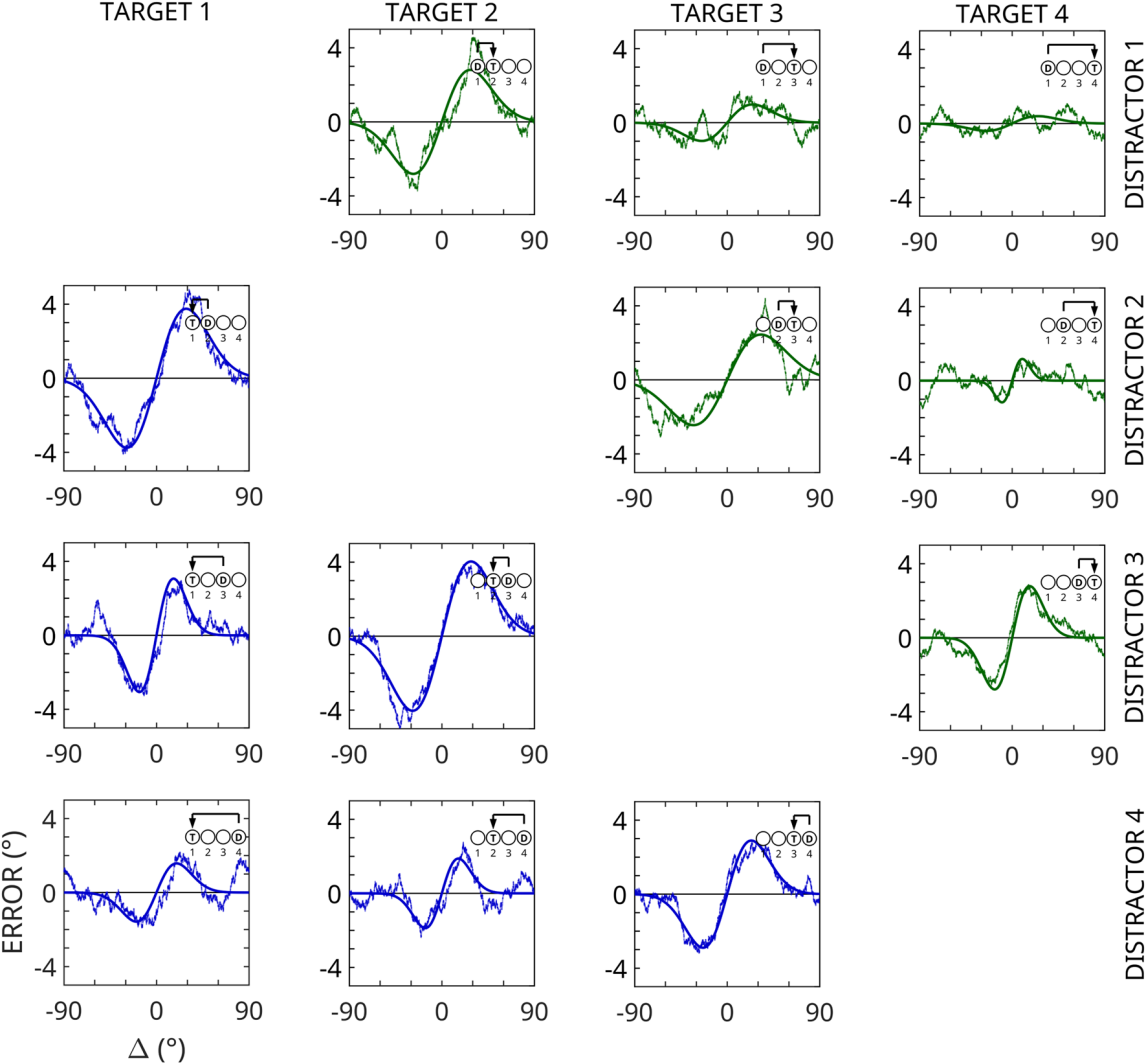
Exploratory results on the combined datasets. Influence of distractor orientation on target orientation report, for each target-distractor combination. The ragged lines represent a moving average of response errors (i.e., response bias; on the y-axis) sorted by the orientation difference between target and distractor (i.e., distractor difference; on the x-axis). The insets in the top right of each panel indicate the analyzed target-distractor combination. The smooth lines depict a fitted DoG function.

**Figure 6:**
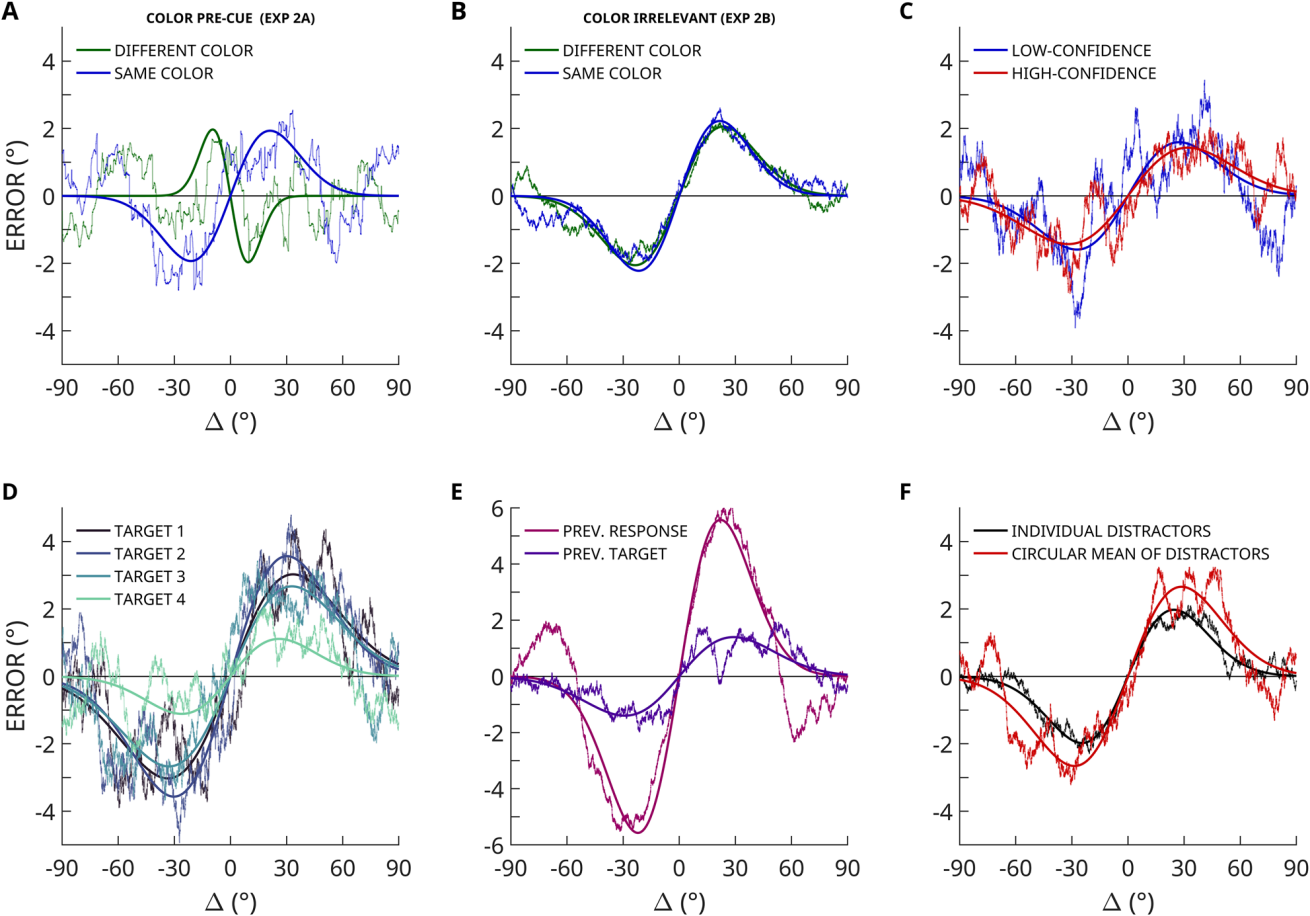
Exploratory results. (**A**) Response bias when target and distractor had the same vs. different colours. The colour of the test item was pre-cued (Experiment 3A). (**B**) Response bias when target and distractor had the same or different colours, but colour was task irrelevant (Experiment 3B). (**C**) Response bias magnitude for data points in which observers rated their responses with low-or high-confidence (Experiment 2). (**D**) The influence of the three distractors (circular mean) on each of the four targets (Experiments 1, 2, and 3B). (**E**) The influence of the target and response from the previous trial on the response of the current trial (Experiments 1, 2, and 3B). (**F**) The response bias toward all distractors combined (i.e., circular mean) compared to individual distractors (Experiment 1, 2, and 3B). In all graphs, the ragged lines represent moving averages, and the smooth lines represent a fitted DoG function.

**Figure 7:**
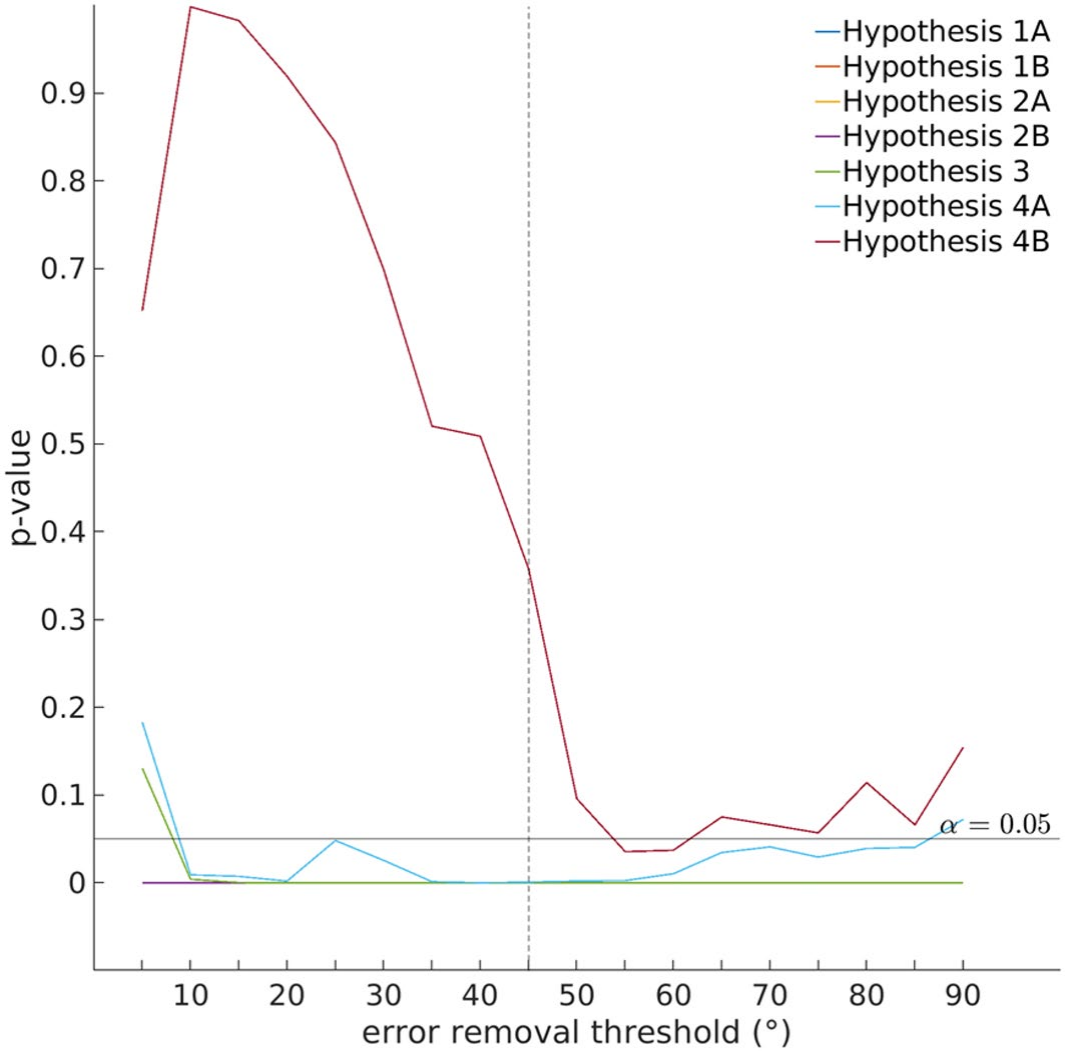
Results for the pre-registered hypotheses for different error removal thresholds (see “Methods” section—“Swap errors”), as a test of robustness of results to analysis choices. With the exception of Hypothesis 4B, results were not sensitive to this parameter. The black horizontal line indicates *p* = 0.05 and the dashed vertical line indicates the results reported here. Note that most result lines overlap at *p* = 0, indicating that the results hold at the *p* < 0.0001 level. Error removal threshold 90 refers to no trials being removed.

The approach of this study was as follows: first, we conducted an exploratory Experiment 1 (N = 43); modelling work on the data of Experiment 1 led us to generate three main hypothesis (Hypotheses 1, 2, and 3, each outlined in a separate paragraph below), which were preregistered at the Open Science Framework (https://osf.io/ytfm9) prior to data collection; these preregistered hypothesis were then tested in an independent data set (Experiment 2; N = 148). Hypothesis 4 was theory driven and pre-registered, but not based on prior modelling work. All analyses reported in the first part of the results section were so-called confirmatory analyses, and pertain to the data set of the pre-registered Experiment 2 (which was virtually identical to Experiment 1; see Fig. 1B). Throughout the Results section (unless otherwise specified), p-values reflect the probability of a Type 1 error, estimated by comparing the observed data to 10,000 generated null-distributions (i.e., permutation tests; see “Methods” section for details). Histograms of response errors for the different experiments and conditions are displayed in Fig. 2. Results are displayed in Figs. 3, 4, 5 and 6. The amplitude and width of the respective tuning functions is summarized in Table 2. Statistical results as a function of researchers’ degrees of freedom are depicted in Fig. 7.

**Table 2:** Amplitude and width of all fitted bias functions.

Dataset	Figure	p (model)	amplitude	width
Experiment 2, forward bias	Figure 3A	< 0.0001	2.04	0.0289
Experiment 2, backward bias	Figure 3A	< 0.0001	1.91	0.0280
Experiment 2, high-noise distractor	Figure 3B	< 0.0001	2.00	0.0307
Experiment 2, low-noise distractor	Figure 3B	< 0.0001	1.99	0.0272
Experiment 2, high-noise target	Figure 3C	< 0.0001	1.82	0.0312
Experiment 2, low-noise target	Figure 3C	< 0.0001	2.15	0.0268
Experiment 2, distance 1	Figure 3D	< 0.0001	2.86	0.0254
Experiment 2, distance 2	Figure 3D	< 0.0001	1.39	0.0444
Experiment 2, distance 3	Figure 3D	< 0.0001	0.63	0.0386
Experiment 3A, different color	Figure 6A	< 0.0001	-1.95	0.0747
Experiment 3A, same color	Figure 6A	< 0.0001	2.15	0.0311
Experiment 3A, different color [model-free]	Figure 6A	0.0002	-0.81	n/a
Experiment 3A, same color [model-free]	Figure 6A	< 0.0001	1.72	n/a
Experiment 3B, different color	Figure 6B	< 0.0001	2.06	0.0300
Experiment 3B, same color	Figure 6B	< 0.0001	2.22	0.0326
Experiment 2, low-confidence	Figure 6C	< 0.0001	1.59	0.0259
Experiment 2, high-confidence	Figure 6C	< 0.0001	1.43	0.0222
Experiment 1 + 2 + 3B, target 1	Figure 6D	< 0.0001	3.03	0.0211
Experiment 1 + 2 + 3B, target 2	Figure 6D	< 0.0001	3.57	0.0234
Experiment 1 + 2 + 3B, target 3	Figure 6D	< 0.0001	2.67	0.0215
Experiment 1 + 2 + 3B, target 4	Figure 6D	< 0.0001	1.11	0.0271
Experiment 1 + 2 + 3B, prev. response	Figure 6E	< 0.0001	5.58	0.0321
Experiment 1 + 2 + 3B, prev. target	Figure 6E	< 0.0001	1.40	0.0243
Experiment 1 + 2 + 3B, individual distractors	Figure 6F	< 0.0001	1.59	0.0259
Experiment 1 + 2 + 3B, circ. mean of distractors	Figure 6F	< 0.0001	1.43	0.0222

### Hypothesis 1: Working memory representations are biased by both preceding and succeeding items

If response biases such as serial dependence can arise during memory maintenance, then information encountered *after* memorization (and after target offset) should influence target report as well. We found that orientation reports were not only biased by items that preceded the target (forward bias, consistent with the classic serial dependence effect; *p* < 0.0001; Fig. 3A), but also by items that succeeded the target (backward bias; *p* < 0.0001; Fig. 3A). This clearly demonstrates that response biases in this paradigm arise during working memory maintenance, because the backward bias can only emerge after the target stimulus was removed from the screen. Surprisingly, the magnitude of the backward response bias was larger than that of the forward bias (*p* < 0.0001; difference between conditions; exploratory analysis). All serial positions were included in this comparison, e.g., in a trial where the first item was the target, the bias from distractors in position 2, 3, and 4 was examined.

### Hypothesis 2: Distractors in both close and far temporal proximity to the target induce a response bias

If the bias arises in memory, not just stimuli immediately preceding and succeeding the target item, but stimuli in the sequence that are one or two items removed from the target, should influence target report as well. The data show that both close and far distractors biased target report (*p* < 0.0001 temporal distance 1; *p* < 0.0001; temporal distances 2 and 3), providing strong evidence that not just distractor orientations observed immediately preceding or succeeding the target item induce a response bias, but also target items further away in time.

### Hypothesis 3: Distractors in closer temporal proximity induce a stronger response bias

We also expected distractors closer in time to the target to induce a stronger bias compared to distractors that are further from the target, which is indeed supported by the data. A permutation test with shuffled condition labels revealed a significant difference in bias magnitude between neighbouring and more distant distractors (*p* < 0.0001). Figure 3D displays the response bias for each of the three temporal distances separately.

### Hypothesis 4: Stronger bias for less reliable targets and more reliable distractors

One functional benefit of generating an averaged representation from multiple observations could be noise reduction: averaging multiple noisy orientation estimates should lead to a less noisy orientation representation. Less reliable representations should be weighted less, and more reliable representations should be weighted more. Specifically, low-noise distractors should influence target report less than high-noise distractors (Hypothesis 4A), and low-noise targets should be more strongly influenced by distractors than high-noise targets (Hypothesis 4B). The data revealed a difference in bias magnitude between low-noise and high-noise distractors (*p* = 0.0014; Fig. 3C), but not between low-noise and high-noise targets (*p* = 0.3667; Fig. 3B). This finding shows that memory averaging exerts a stronger influence on memory reports, when the non-target item contains less noise; that is, when averaging would be most beneficial to behaviour.

## Exploratory analyses

Unless otherwise specified, the analyses reported below are conducted on the combined data sets of Exploratory Experiment 1, Pre-registered Experiment 2, and Experiment 3B (total N = 246), all of which required observers to retroactively report one out of four sequentially presented memory items. The amplitude and width of the respective tuning functions is summarized in Table 2 above.

### All items in working memory are influenced by every other item

If behavioural responses are the result of a functionally weighted average representation obtained during separate episodes of sensory input, then the reproduction of a memorized item should be influenced by all similar items in working memory. That is, most (if not all) items in the sequence should influence the representation of every other item in the sequence, provided they are stored in memory. Indeed, all combinations of distractor and target produced a significant response bias (all *p* < 0.0001; Fig. 5), appearing as attraction between items with similar orientations. This is also visualised in a 3D surface plot as the combined influence of distractors preceding and succeeding the target (Fig. 4).

### Opposite effects of task-relevant and task-irrelevant distractors

A functionally weighted average should include only those items that are task relevant. If the response bias reported here reflects a functionally beneficial principle, rather than an artefact of the system, we would expect that only behaviourally task-relevant items in memory influence target report. To investigate how task-relevance impacted the response bias, we conducted Experiments 3A and 3B, in which each orientation in the sequence was presented in one of two colours. Distractors could have the same or a different colour than the target. We expected that only similarly coloured distractors would induce a bias because observers would know that differently coloured items could not be the target.

In Experiment 3A observers were pre-cued with one of the two colours and instructed that only targets of the pre-cued colour would be tested. By doing so, we manipulated the task relevance of the individual items in a sequence. We found a response bias consistent with the data pattern observed in Experiments 1 and 2 when the distractor was the same colour as the target (Fig. 6A); target reports were attracted toward similar distractors. In contrast, when target and distractor were of a different colour, we observed a reversal of this effect: similar distractor orientations caused a repulsion of the target report (*p* < 0.0001). The difference between these two conditions was significant (*p* < 0.0001). We additionally confirmed this result in a model-free analysis by taking the mean error for delta > 0 plus the sign-flipped mean errors for delta < 0 as a parameter-free, conservative estimate of response bias. This analysis confirmed the attraction bias for the same color condition (*p* < 0.0001), the repulsion bias for the different color condition (*p* = 0.0002), and the difference between these conditions (*p* < 0.0001) using permutation tests with shuffled condition labels. The repulsive effect observed for dissimilar colours shows that task-irrelevant items—which observers did not need to memorize because they would not be tested—were nonetheless stored in memory, and influenced responses in a direction opposite to that of task-relevant working memory items.

The qualitatively different biases of task-relevant and task-irrelevant distractors observed in Experiment 3A might have been caused simply by the visual dissimilarity of the differently coloured items. To test this, we conducted Experiment 3B, in which observers were presented with the same coloured orientations as in Experiment 3A, but the target colour was not pre-cued. Hence, the stimuli differed in colour, but not in task relevance. In contrast to Experiment 3A, no difference in response bias was found between differently and same coloured target-distractor pairs (*p* = 0.3605; Fig. 6B). Instead, the response bias was present when targets and distractors were of same colour (*p* < 0.0001) and when they were of different colours (*p* < 0.0001). Together, these results indicate that dissimilarities between distractor and target on a task-relevant, but not task-irrelevant feature dimension reduce and even reverse the response bias.

### Self-reported confidence

After each trial in Experiment 2 observers rated their confidence in the orientation report that they just provided. We expected that orientation reports on low-confidence trials (“Not quite sure.”) would be influenced more strongly by distractors than on high-confidence trials (“I am reasonably sure.”). Self-reported guesses (“Pure guess, I have no idea.”) were excluded from this analysis. The data only revealed a non-significant trend in the expected direction (*p* = 0.3998; comparison between conditions; Fig. 6C).

### Targets earlier in the sequence are influenced more strongly

Target reports for items earlier in the sequence are characterized by a decreased memory recall precision. This is to be expected as representations decay over time and become more variable as additional information is encoded. Thus, targets earlier in the sequence should be more strongly influenced by distractors. This is exactly what we found. Comparing the response bias magnitude in the first and last target in the sequence (using datasets from Experiment 1, 2 and 3B) reveals a significant difference in the predicted direction: the first target in the sequence was more strongly influenced than the last target in the sequence (*p* < 0.0001; difference between conditions; Fig. 6D). This finding adds support for Hypotheses 4A, indicating that the less reliable, earlier targets were influenced more strongly than more reliable, recent targets.

### Previous targets and responses bias current responses

In the previous sections we focused on within-trial effects, that is, the influence of representations currently held in working memory on target report. Conveniently, the present paradigm allows to concurrently examine the influence of the previous trial on current target report. Previous target response strongly biased current responses (*p* < 0.0001). This represents a replication of the classic serial dependence effect (Fig. 6E). The tuning curve of the response bias from previous trial responses (between-trial effect) bears remarkable similarity to that of the within-trial effects reported in the rest of this study (Fig. 6F). In addition, the veridical (i.e., displayed) orientation of the target in the previous trial also biased current responses (*p* < 0.0001), but to a lesser extent than the previous responses (*p* < 0.0001; difference between conditions).

### Circular mean of distractor orientations biases responses

A weighted averaging process within visual working memory would predict that the circular mean of the three distractors would bias responses more strongly than each distractor alone. This is precisely what we found (Fig. 6F). Combining the datasets of Experiment 1, 2 and 3B, comparing the bias magnitude when analysing the influence of the circular mean of the three distractors in each trial (*p* < 0.0001) to the bias magnitude when analysing each distractor individually (*p* < 0.0001) revealed a significant difference (*p* < 0.0001).

## Discussion

We investigated how the reproduction of a memorized target was systematically biased towards concurrently memorized distractors. Target report was consistently influenced by orientations that were shown *before* the target in the sequence as well as orientations shown *after* the target was no longer visible. We observed that any item currently held in working memory influences target report if it is sufficiently similar to the target on a task-relevant feature dimension. Responses were biased less when the target representation was more reliable (less noisy), later in the sequence (more recent) or closer to the target in time. We propose that such response biases in memory recall may reflect a functionally weighted average of all relevant stimuli that were observed across multiple episodes of sensory input.

### Memory reports are biased by previously and subsequently memorized stimuli

In addition to a “forward bias” (i.e., similar to classic serial dependence), we also observed a “backward bias”. Distractors biased target response even when stimuli were presented after perceptual and encoding stages of the target were completed (Hypothesis 1). It has been debated whether response biases such as serial dependence arise during perception, that is, whether it is the perception of a new stimulus that is biased^19,28,29,36–39^, or whether the bias arises during post-perceptual stages, such as memory maintenance^4,18,26,40,41^. It is reasonable to assume that mechanisms such as serial dependence are implemented at different processing stages^41,42^. While the present results do not exclude the possibility that additional biases emerge during stimulus encoding, we do establish that representations can be biased *after* stimulus encoding, that is, during memory maintenance or retrieval.

### The memory report bias is weighted by similarity, reliability, and task-relevance

One crucial benefit of integrating memory representations could be noise reduction. Behavioural effectiveness crucially depends on a functionally optimal representation of world states. Any estimate based on a functional integration of several observations necessarily is more reliable than a single observation. Recent work has shown that sequentially presented stimuli are simultaneously represented in brain activity even without explicit memorization instructions^3^. Such concurrent storage potentially allows the visual system to combine multiple observations to generate an averaged representation that is more accurate than any single observation alone. However, it would also be expected that not all observations are weighted equally. For example, a behaviourally beneficial memory integration mechanism would be expected to integrate only those representations that are likely to have been sampled from the same object. Indeed, it became evident in the present data that target report is consistently attracted by *similar* orientations in memory. Comparable effects of target-distractor similarity were previously reported in the context of response biases^43^, visual search^44^, as well as in studies investigating across-trial serial dependence effects^17^.

Another expected attribute of behaviourally beneficial memory integration would be that more *reliable* information is weighted more strongly than unreliable information. Our data show that less noisy, more reliable distractors exhibited a stronger influence on target responses than more noisy, less reliable distractors (Hypothesis 4B). There was no reliable evidence that noisy, less reliable targets were more strongly biased. In line with our current findings, it has previously been demonstrated^1^ that the influence of unreliable on more reliable representations was smaller than the influence of reliable on unreliable representations. Furthermore, it has recently been demonstrated that uncertainty is maintained and used in working memory and reflects the precision of memory item representations^45^. Our data extend these findings in showing that the magnitude of the bias is influenced mainly by the reliability of the distractor.

When information from multiple observations is combined, it would be beneficial to weigh these observations according to their *relevance* to the behavioural goal. It has previously been shown that when multi-feature objects had to be remembered, task-relevant features induced a larger attractive bias than task-irrelevant features^24^. In the present study, when presented with orientations that are randomly coloured in one of two colours, observers’ responses were biased toward all other distractor orientations, irrespective of their colour. However, when observers were additionally presented with an informative pre-cue before the item sequence, indicating the colour of the target, orientations with non-cued colours created a repulsive bias, exactly opposite to that of cued items (Fig. 6A). The presence of a significant, reversed response bias indicates that un-cued distractors were encoded in memory, despite not being relevant for the task at hand, and that their representations were actively distinguished from the target. In a recent study^43^, the authors found an attractive bias toward a second stimulus when a perceptual comparison is made. However, they also found an attractive bias (although attenuated) when the comparison stimulus was to be ignored. It is unclear if this and the present study can be compared given the very different memory demands. Together, these findings are indicative of an active goal-directed averaging mechanism, rather than a passive process.

### Interpreting the magnitude of the observed bias

One could wonder whether biases of a few percentages, as observed in our study and similar studies, are sufficient for (1) providing a substantially more accurate representation of any individual item, and (2) for gluing perceptual snapshots together to form a smooth and coherent percept of the world. It should be noted, however, that in our study we explicitly instructed to report on a single, individuated stimulus in order to investigate how individual memory reports are influenced by concurrently held memoranda. The fact that we observed any bias at all, despite the explicit instruction to report individual items, speaks to the existence of an obligatory component of memory integration. Importantly, the bias was not a uniform attraction or repulsion, as would be expected from a mere side-effect of a memory encoding mechanism but followed a tuning curve comparable to results found in investigations of serial dependence. Future research could identify the underlying neurophysiological mechanisms and explore the connection between individual item biases measured during recall and averaged representations of memory contents. A paradigm could be employed that more closely resembles the process of taking perceptual snapshots in the wild, for instance using continuous stimuli that are sampled during consecutive fixation periods.

Another point of caution pertains to the occurrence of swap errors, in which the observer reports the wrong (un-cued) item. In such a swap trial, the response would be coded as a 100% bias towards the distractor. When averaging data across multiple trials, a consistent attractive bias (of, say, 2% on each trial) is indistinguishable from swap errors on 2% of all trials. This is an issue that is almost entire ignored by the response bias literature. Nevertheless, steps can be taken to reduce the influence of swap errors. In the present study we found that removing trials with large response errors (i.e., here larger than 45°) serves as a simple, yet effective way of removing a large percentage of swap errors, at least for large deltas, without causing systematic distortions of the data. For small deltas it becomes increasingly impossible to distinguish on a trial-by-trial basis between a swap error and a true bias and likely still inflates the estimates of individual bias functions. Importantly, swap errors do not inflate the differences between conditions, that is, the differences in the amplitudes of the respective bias functions. This is because swap errors would be equally likely to occur in either condition (see Fig. 10). While there likely is no perfect way of accounting for swap errors, we have validated our approach through simulations (Figs. 9 and 10) and verified the robustness of our results for different error removal thresholds (Fig. 7). Moreover, it has been shown that when observers are required to memorize two different spatial frequencies, they make a disproportionate number of false alarms to intermediate spatial frequencies^8^. This cannot be easily explained by swap errors, and likely reflects within-trial averaging of the memory content.

### Within-trial versus between-trial biases in memory report

When a previous observation influences a current observation, it must have been stored in some form. Thus, any response bias observed between items that are not simultaneously presented necessarily involves memory. Serial dependence (a response bias that arises between trials) is often described as a very similar principle as memory averaging: by taking into account previously encountered information, presently encountered information can be more reliably estimated. Here, we consider the idea that between-trial biases—such as serial dependence—might be one expression of a more general process of memory averaging over time (or temporal smoothing), akin to the within-trial biases observed here. In contrast to typical serial dependence paradigms, participants in our study were explicitly instructed to memorize all stimuli in a sequence. In typical serial dependence paradigms, participants are only instructed to report the last seen stimulus, such that there is no explicit requirement to memorize the preceding stimulus. Could it be that similar mechanisms underlie between-trial biases without explicit memory instructions (e.g., the classic serial dependence effect), and within-trial biases with explicit memory instructions (as observed in the current study)? For serial dependence to occur, information from the previous stimulus must have lingered in memory in some form despite no instruction or benefit of memorizing these stimuli beyond the trial. As such, both within and between-trial biases require memory storage of the non-reported stimuli. In our exploratory analysis of between-trial biases, we showed that the response to the current target stimulus was also biased toward the response in the previous sequence, although participants no longer needed to keep these previous items in memory. Moreover, the tuning function of this between-trial attractive bias was strikingly similar to that of the within-trial biases (Fig. 6C), demonstrating that an explicit memorization instruction is not required to induce a response bias similar to the one observed with an explicit memorization instruction. This suggests that between-trial biases and within-trial biases might be two expressions of the same underlying mechanism of memory-averaging. Other researchers, however, have observed qualitatively different within-trial and between-trial biases. For instance, in a study where participants concurrently memorized two grating orientations on each trial, orientations presented in the same trial mainly affected perception, whereas orientations presented in previous trials mostly affected memory reports^9^. Also, in a study where participants concurrently memorized two motion directions on each trial, memory reports were attracted toward motion directions of the previous trial but repelled from motion directions in the current trial^5^. Interestingly, in these studies, only two stimuli were presented within a given trial, whereas many stimuli were presented across trials, making within-trial and between-trial biases difficult to compare. It could be argued that when only two items are memorized, an appeal is made on mechanisms that promote segregation of the two items, since the task is to report either one or the other item (this could also be explained in terms of reciprocal inhibition between two concurrent memory representations^46^). Instead, when more items are memorized—and storage of each individual item becomes increasingly resource-costly—an appeal is made on mechanisms that promote integration^35^. From another perspective, it could be argued that when only two stimuli are memorized, more attentional resources are allocated to each item than when more stimuli are memorized, which could increase (or create) a repulsive bias^13^. Indeed, using four consecutive stimuli, we observed an attractive within-trial bias, whereas others observed a repulsive within-trial bias using only two stimuli^4,5,13,33,46^. The within-trial (and between-trial) biases observed in the current study strongly resemble the between-trial biases reported in the literature, such as the classic serial dependence effect. This suggests that, under comparable circumstances, similar mechanisms might underlie within-trial and between-trial biases.

## Conclusion

In summary, our findings show that response biases observed in the present study—and probably also many responses biases from the short-term memory literature—may reflect different expressions of a generic mechanism of functional memory integration, in which individual representations are weighted according to their similarity on a task-relevant feature dimension and their reliability. This process may capitalize on generic mechanisms of perceptual averaging, akin to those governing ensemble perception^47,48^, but applied to sequentially presented items that occur in close temporal proximity. We speculate that such functionally weighted averaging of memory content may facilitate noise reduction in the face of sensory uncertainty and support the stability of perception and behaviour. The visual system effectively makes use of the statistical regularities and continuity present in the real world, on both larger and shorter timescales. As the present results show, working memory appears to play a key role in the implementation of these principles.

## Methods

Results reported here are based on four experiments. Modelling work based on Experiment 1 formed the basis of the pre-registered Hypotheses 1, 2 and 3, while pre-registered Hypothesis 4 was theory driven rather than based on prior modelling work. Experiment 2 was designed to test the four pre-registered hypotheses (Pre-registered Results section). In addition, Experiments 3A and 3B were conducted to address further questions of interest that were not pre-registered (“Exploratory analyses” section). The pre-registration protocol can be found at https://osf.io/ytfm9 and data is available at https://osf.io/embcf/.

### Participants

Observers were recruited via the online research platform Prolific (http://www.prolific.co) and were compensated for their time with 8, euro/hour. Inclusion criteria were set to age 23–50 years, no literary difficulties, completed education level of at least BA/BSc, no psychiatric medication use, normal or corrected to normal vision, fluency in English, no daily impact of mental illness, less than 10 units of alcohol consumed per week, at least 95% approval rating on Prolific and 10 minimum previous experiment participations. All observers provided informed consent. The study was approved by the local ethics committee at Utrecht University and all methods were carried out in accordance with relevant guidelines and regulations.

Sample sizes were chosen for pragmatic (i.e., financial) reasons. From a total maximum, the number of observers was allocated to each experiment according to priority. In Experiment 1 we aimed for 50 observers, in Experiment 2 for 150 observers (as it was the most important and confirmatory experiment), in Experiment 3A for 80 observers and in Experiment 4B for 50 observers. As a result of exclusions, subsequent opening of experiment slots, and anticipated failed participations in the online experiment platform, the actual number of observers was somewhat variable. Future studies would benefit from choosing sample sizes through a priori power calculations when effect sizes are known. Observers providing data with flat error distributions (indicating random responses) were excluded from analysis (Experiment 1: 19, Experiment 2: 10, Experiment 3A: 2, Experiment 3B: 17), as well as observers who failed an attention check (a simple question that could only be answered correctly by viewing the entire instruction video; Experiment 2: 11, Experiment 3B: 6). In addition, and according to the pre-registered protocol, 3 observers with extremely similar error distribution histograms between the first and the last target were excluded from Experiment 2 due to suspected cheating as it is highly unlikely that observers were able to perform equally well for the first (most difficult) and last (easiest) target. In addition, due to a file copying error, two observers’ datasets were lost. After exclusion, 43 observers participated in Experiment 1, 148 observers participated in Experiment 2, 84 observers participated in Experiment 3A, and 55 observers participated in Experiment 3B. In Experiment 1, observers completed on average 159 ± 43 (mean ± STD) trials (range 39–213), 6822 total trials. In Experiment 2, observers completed on average 103 ± 26 trials (range 41–175), 15,297 total trials. In Experiment 3A, observers completed on average 267 ± 64 trials (range 118–334), 22,440 total trials. In Experiment 3B, observers completed on average 131 ± 26 trials (range 50–184), 7196 total trials.

### Stimuli and materials

All stimuli were displayed at the centre of the screen in sequence. The exact size and colour of stimuli varied according to the screen of the participant, which was required to be at least 719 px in height. Similarly, timing inaccuracies associated with online experiments likely resulted in some variance in presentation times (in the order of up to tens of ms). It is highly unlikely that the variance in stimulus presentation would have qualitatively influenced the pattern of results reported here. There was always only one stimulus on the screen at a time and all manipulations were within-subject (except for the contrast between Experiments 3A and 3B). While such variation might have increased measurement noise, this would only result in more conservative test outcomes and any variation in presentation parameters would make the experiment more representative of the natural world. The screen background was set to 50% grey (RGB 127 127 127). Memory stimuli consisted of oriented line gratings (orientations), with a Gaussian envelope with a standard deviation of 80 pixels and a spatial frequency of 75 pixels per cycle. The mask, which was displayed after each orientation (an orientation patch, see Table 1), consisted of procedurally generated noise with a spatial frequency distribution matching that of the orientation presentations, but which contained no orientation information. In Experiments 3A and 3B, each orientation could be displayed as randomly drawn from one of two colours, cyan (RGB: 0 255 255) and yellow (RGB: 255 255 0). In Experiment 3A, an informative pre-cue was presented before the orientations, which consisted of the word “BLUE” or “YELLOW” at the centre of the screen. In Experiment 2, the noise manipulation was induced through a linear combination of the orientation stimuli and smoothed white noise. Each orientation could be displayed as randomly drawn from one of two noise levels: high-noise, with a stimulus contrast of 0.05 and a noise contrast of 0.4, or low-noise, with a stimulus contrast of 0.2 and a noise contrast of 0.3.

### Procedure

Each experiment began with an instruction video, explaining the experiment to the observer, who were in addition instructed to find a quiet, dark, and clean space to conduct the experiment. For Experiments 2 and 3, at the end of the video a hint was given that the subsequent questionnaire item “What is 25 + 13?” should be answered with “19”. This was done to test whether an observer actually watched the instruction video. Subjects who responded anything other than “19” (including the correct outcome of the equation, “38”) were excluded from analysis. This was followed by a practice round of 11 trials. After each practice trial, participants were given feedback on the magnitude of the response error in that trial. After the practice round, the main experiment began. After every 10 trials, feedback was given indicating the average response error over these last 10 trials. After 20 and 40 min experiment time (Experiment 1 and 2) or after 25 min (Experiment 2), observers were invited to take a short break. Experiments 1 and 3 terminated after 60 min total time, and Experiment 2 terminated after 45 min.

#### Experiment 1

Each trial began with a blank screen for 500 ms, followed by four repetitions of the sequence: orientation for 300 ms, blank for 200 ms, noise mask for 100 ms, blank for 750 ms and a cue for 200 ms. The cue indicated which of the four orientation targets the observer should report: − 4 for the first orientation, − 3 for the second, − 2 for the third and -1 for the most recent orientation in the sequence. Following the cue was a blank for 750 ms, after which a randomly oriented line was presented, which could be rotated using the mouse to indicate which orientation was remembered. A mouse click confirmed the response and the next trial began. See Fig. 1A,B for a visual depiction of a trial sequence in each experiment.

#### Experiment 2

Each trial began with a blank screen for 500 ms, followed by four repetitions of the sequence: orientation for 200 ms on average (randomly drawn from a range between 140 and 260 ms, in steps of 8 ms), blank for 100 ms, noise mask for 50 ms, blank for 500 ms and a cue for 200 ms. Each orientation could either contain high visual noise or low visual noise. The cue indicated which of the four orientation targets the observer should report: 1 for the first orientation, 2 for the second, 3 for the third and 4 for the most recent orientation in the sequence. Following the cue was a blank for 750 ms, after which a randomly oriented line was presented, which could be rotated using the mouse to indicate which orientation was remembered. A mouse click confirmed the response. After responding observers were asked to rate their confidence in the accuracy of their response by pressing 1, 2 or 3 on the keyboard, corresponding to “Pure guess, I have no idea.”, “Not quite sure.” And “I am reasonably sure.”, respectively. These hints were displayed on screen, next to the corresponding number. After observers gave their confidence response the next trial began.

#### Experiment 3A and 3B

Each trial began with a blank screen for 500 ms, followed by a pre-cue in Experiment 3A, consisting of the written-out words “BLUE” or “YELLOW” in black, which was omitted in Experiment 3B, followed by four repetitions of the sequence: orientation for 300 ms, blank for 200 ms, noise mask for 200 ms, blank for 750 ms. Each orientation and corresponding mask were colourized in one of two colours: cyan (blue) or yellow. Participants were explicitly instructed that whenever “YELLOW” was pre-cued, only yellow targets would be cued for report, and whenever “BLUE” was pre-cued only cyan targets would be cued for report. This was followed by the recall cue for 200 ms, indicating which of the four orientations the observer should report: − 4 for the first orientation, − 3 for the second, − 2 for the third and − 1 for the most recent orientation in the sequence. Following the cue was a blank (750 ms), after which a randomly oriented line was presented, which could be rotated using the mouse to indicate which orientation was remembered. A mouse click confirmed the response and the next trial began.

### Swap errors

**Figure 8:**
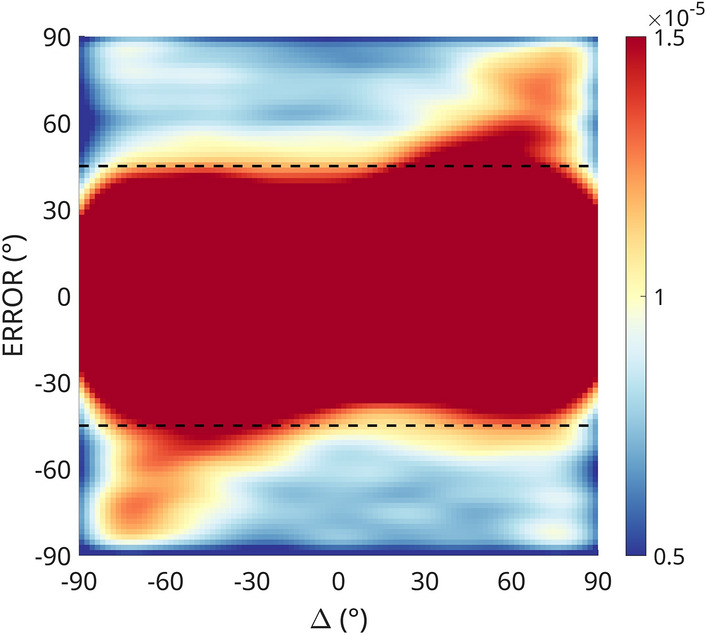
Kernel density plot of all data points from Experiment 1, 2 and 3B. Swap errors can be observed along the identity line (diagonal from -90/-90 to 90/90) as increased density. The dashed lines indicate the error removal threshold used in the present study.

The present data sets (and very likely all data sets from studies investigating response bias or serial dependence) contain a significant amount of swap errors. These are trials in which the observer reported the distractor orientation instead of the target. Swap errors can be recognized as responses that follow the identity line (e.g., an error of + 30° when the distractor deviated + 30° from the target), as seen in the density plot in Fig. 8 below, which demonstrates the presence of swap errors in the present dataset.

**Figure 9:**
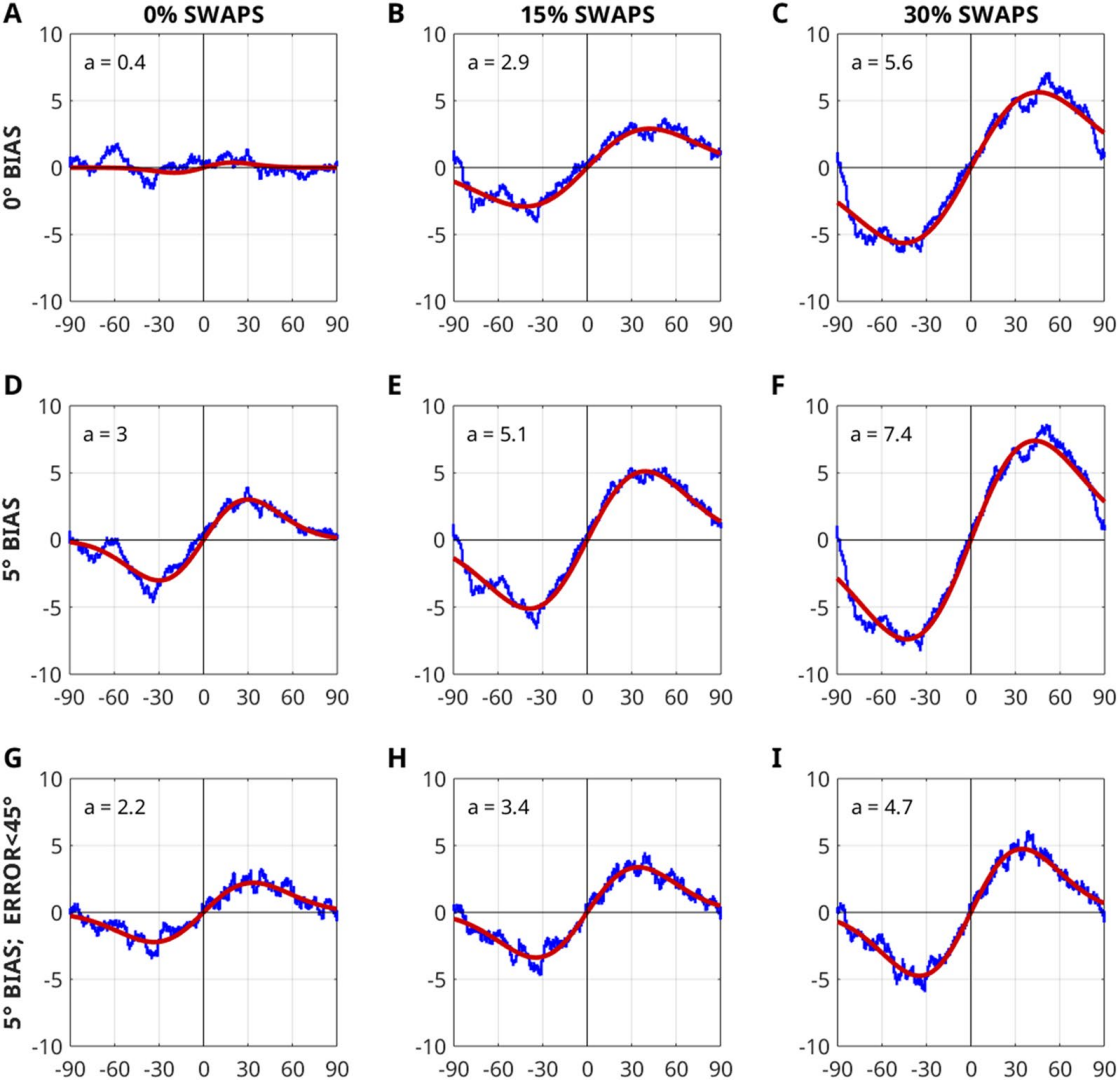
Simulated data illustrating how swap errors can induce a data pattern similar to a response bias. In this simulation, response error is modelled by a Von Mises distribution with a concentration parameter k = 4. 10% of data points are modelled as random guesses. In the top row (**A**–**C**) no bias is simulated. In the middle row (**D**–**F**), in addition to swap errors, a 5 deg bias is added to the data. In the bottom row (**G**-**I**), large errors (> 45°) are removed from the data prior to analysis.

Across trials, swap errors can induce a data pattern that closely resembles the typical derivative of Gaussian (DoG) function of an attractive response bias, even in the absence of such a bias in individual responses. Figure 9 below demonstrates this problem by showcasing results from simulated data (e.g., the data of panel B actually contains a response bias but is indistinguishable from panel D in which only swap errors occur and no response bias is present). A simple and straightforward solution to correct for swap errors is to remove large errors (here errors larger than 45°). This removal is a deviation from the pre-registered protocol. It is illustrated in Fig. 9H, which after removal shows as a comparable bias function as that observed in the swap-free data set (Fig. 9D). This procedure has the advantage of not inducing artificial biases in the data set but does not disentangle bias from swaps in data points with small deltas, and it is unlikely that any procedure will be able to. Future research should attend to this general and largely un-addressed problem in the field of response bias and serial dependence.

**Figure 10:**
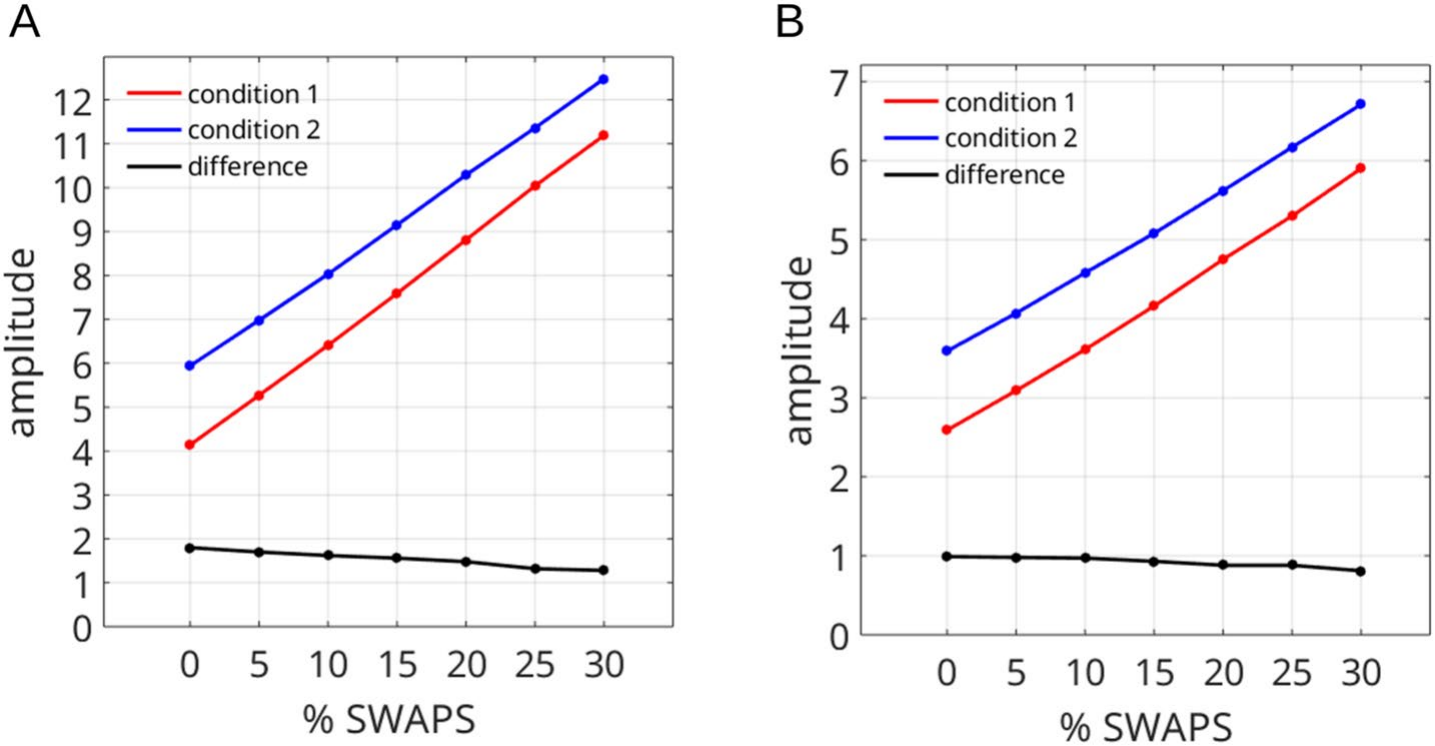
Simulated data, illustrating the behaviour of differences between conditions (differences in DoG amplitude) under the presence of different amount of swap errors in the dataset. (**A**) Full data set (as in Fig. 9). (**B**) trials with response errors larger than 45° removed. While individual amplitudes were over-estimated in the presence of swap errors, differences between conditions were not, and even slightly under-estimated, making the results conservative.

Importantly, when simulating different conditions under the presence of swap errors (e.g., high versus low noise targets), it becomes clear that while the presence of swap errors does inflate the magnitude of the measured bias it does not inflate differences between conditions (i.e., differences between amplitudes in fitted DoG functions), and even slightly underestimates them. This is illustrated in Fig. 10.

### Data analysis

Each trial contained three “data points”: one for each target-distractor combination. That is, there were three possible items in a trial that could bias report of the fourth item, the target. Each data point was represented by the angular difference between target orientation and reported orientation (response error), as well as the angular difference between distractor and target. In order to estimate the presence and magnitude of a response bias, response errors were sorted by the difference between target and distractor (∆). A derivative of gaussian (DoG) function was then fitted to this data through least-squares optimization (Eq. 1).

1 $$f\left(x\right)={xawce}^{-{\left(wx\right)}^{2}}$$

where $x$ is the relative orientation of the distractor, $a$ is the amplitude, $w$ is the curve width, and $c$ is the constant $\sqrt{2}/{e}^{-0.5}$. To ensure convergence, bounds were set to $\alpha$ = [− 10, 10], $w$ = [0.01, 0.08]. Starting values were set to $a$ = 2, and $w$ = 0.2.

The model function was fitted to the raw data. To visualize the data, a moving average was computed using the movmean function in Matlab with a symmetric window of size 0.04 * data length. For each experiment, data was combined from all observers into a single vector. Future studies may benefit from a hierarchical per-observer fit, which would ideally require multiple experimental sessions per observer; an approach that is not well suited to an online study.

Statistical analysis was performed through permutation tests. *p*-values were obtained by comparing the amplitude of the best fit on the observed data to the amplitudes of the best fits on 10,000 random permutations of the dataset. These permutations were produced by shuffling target-distractor differences. *p*-values indicate the proportion of shuffled data sets in which the amplitude of the best fit was equal to or larger than that of the ordered (i.e., observed) data set. An effect was considered to be significant at the α = 0.05 level. Differences between conditions were obtained through similar permutation tests, but with shuffled condition labels instead of shuffled target-distractor differences^15,41^.

## Data Availability

Data is available at https://osf.io/embcf/. Pre-registration is available at https://osf.io/ytfm9.
